# An Effective Graph Clustering Method to Identify Cancer Driver Modules

**DOI:** 10.3389/fbioe.2020.00271

**Published:** 2020-04-07

**Authors:** Wei Zhang, Yifu Zeng, Lei Wang, Yue Liu, Yi-nan Cheng

**Affiliations:** ^1^College of Computer Engineering and Applied Mathematics, Changsha University, Changsha, China; ^2^Hunan Province Key Laboratory of Industrial Internet Technology and Security, Changsha University, Changsha, China; ^3^Key Laboratory of Hunan Province for Internet of Things and Information Security, Xiangtan University, Xiangtan, China; ^4^College of Computer Science and Electronics Engineering, Hunan University, Changsha, China; ^5^College of Science, Southern University of Science and Technology, Shenzhen, China

**Keywords:** driver modules, mutual exclusivity, connectivity, functionally similarity, Markov clustering

## Abstract

Identifying the molecular modules that drive cancer progression can greatly deepen the understanding of cancer mechanisms and provide useful information for targeted therapies. Most methods currently addressing this issue primarily use mutual exclusivity without making full use of the extra layer of module property. In this paper, we propose MCLCluster to identity cancer driver modules, which use somatic mutation data, Cancer Cell Fraction (CCF) data, gene functional interaction network and protein-protein interaction (PPI) network to derive the module property on mutual exclusivity, connectivity in PPI network and functionally similarity of genes. We have taken three effective measures to ensure the effectiveness of our algorithm. First, we use CCF data to choose stronger signals and more confident mutations. Second, the weighted gene functional interaction network is used to quantify the gene functional similarity in PPI. The third, graph clustering method based on Markov is exploited to extract the candidate module. MCLCluster is tested in the two TCGA datasets (GBM and BRCA), and identifies several well-known oncogenes driver modules and some modules with functionally associated driver genes. Besides, we compare it with Multi-Dendrix, FSME Cluster and RME in simulated dataset with background noise and passenger rate, MCLCluster outperforming all of these methods.

## Introduction

Cancer research has shown that gene mutation can disrupt specific cellular pathways that drive cancer development (Weinstein et al., 2013). Recently, the rapid development of next-generation sequencing technologies has increased the generation and availability of high-resolution data related to cancer, providing opportunities for the study of cancer genomes (Wood et al., 2007; Cancer Genome Atlas Research, 2008; Tomczak et al., 2015; Zhao et al., 2019). The key task of cancer genomes research is to identify the molecular mutations or drivers. Functionally related driver mutations in the genome, also known as driver modules or pathways, activate the mechanisms by which cancer occurs, triggering cancer, driving cancer progression and giving cancer cells a selective advantage.

Some computational methods and mathematical models have been developed to detect driver gene sets, pathways and modules by using large-scale sequencing data (Hou et al., 2016; Zheng et al., 2016; Yang et al., 2017; Xi et al., 2018; Ahmed et al., 2019; Deng et al., 2019; Zhang and Wang, 2019a; Pelegrina et al., 2020). Existing research show that the members of cancer driver modules often exhibit specific mutation patterns in cancer samples, the most significant characteristic is mutual exclusivity (mutex) which means once one member mutates, the tumor will gain a significant selection advantage, while later mutations in other members will not give the tumor a selection advantage. Most current methods use only mutex to derive the driver pathway or modules, the other properties of the module are not fully considered, such as functionally similarity of members within a module.

Recently, two types of methods for identifying driver modules or gene sets have been proposed: De novo and knowledge-based methods. The De novo methods usually exploit two characteristics from somatic mutation data: high coverage and mutex (Dees et al., 2012; Vandin et al., 2012; Zhao et al., 2012; Babaei et al., 2013; Leiserson et al., 2013; Paull et al., 2013; Jia et al., 2014; Deng et al., 2019; Zhang and Wang, 2019a,b; Dees et al., 2012; Vandin et al., 2012; Zhao et al., 2012; Babaei et al., 2013; Leiserson et al., 2013; Paull et al., 2013; Jia et al., 2014; Deng et al., 2019; Zhang and Wang, 2019a,b). High coverage means that the driver modules or driver pathway covers a large number of samples. Mutex represents that one of driver gene mutations in a pathway are sufficient to interfere with the pathway. For example, Dendrix (Vandin et al., 2012) identifies driver pathways with high coverage and mutex by transforming the problem into a maximum exclusive sub-matrix. MDPFinder (Wu et al., 2015), Multi-dendrix (Leiserson et al., 2013), ComMDP, and SpeMDP (Zhang and Zhang, 2016) figure out the maximum exclusion sub-matrix problem by utilizing the integer linear programming, focus on identifying mutex gene sets. On the other hand, the knowledge-based approaches, in addition to somatic mutation data, other network- and functional phenotype-based data are combined to detect driver pathway or modules (Hua et al., 2013; Babur et al., 2015; Kim et al., 2015; Leiserson et al., 2015; Nambara et al., 2015; Wang et al., 2015; Reyna et al., 2018; La Vecchia and Sebastian, 2020). These approaches can be subdivided according to the optimization objectives in the computational problem, and they are used to define cancer driver modules identification problems. In the methods of Hotnet (Network, 2012), Hotnet2 (Leiserson et al., 2014), Hierarchical Hotnet (Reyna et al., 2018), thermal diffusion is a common feature. Diffusion values are used to extract modules with high connectivity, which are defined by graph connectivity (usually strong connectivity). Other methods, such as MEMo (Ciriello et al., 2012), RME (Leiserson et al., 2015)and FSME Cluster (Liu et al., 2017), use the interaction network and function relation graph to derive the largest group in the similarity graph, and derive the group with largest mutex. Babur et al. (Babur et al., 2015) proposed a seed growth-based method in the network, which uses TCGA data to identify pan-cancer modules, and the method determines the growth strategy based on mutex scores. Dao et al. (Dao et al., 2017) proposed an ILP method, which combined the definition of interaction density and mutex in the module as the optimization target. MEMCover (Kim et al., 2015) and MEXCOwalk (Ahmed et al., 2019) combined mutation data with interaction data to detect mutually exclusive mutant genomes in the same or different tissues.

In this work, we get inspired by these existed methods and present a novel knowledge-based method to identify cancer driver modules (MCLCluster), which combines mutex, functional similarity and connectivity in PPI network, multiple data type is used. Before we compute the mutex, the Cancer Cell Fraction (CCF) is aided to select stronger signals and more confident mutations, then the weighted gene functional interaction network is used to quantify the gene functional similarity in PPI, exploit graph clustering method based on Markov to extract the candidate module. The similarity measure between a pair of genes is defined as PPI network edge weight through taking into account functional similarity and mutex. Cluster filter and permutation test is used to test which cluster to be driver modules. We compare it with those of three representative approaches [Multi-Dendrix (Leiserson et al., 2013), FSME Cluster (Liu et al., 2017), and RME (Leiserson et al., 2015)] on simulated dataset with background noise, MCLCluster outperform all of these methods. Unlike most of presented approaches to discover driver modules with mutually exclusive between all gene pairs, MCLCluster does not necessarily identify complete exclusivity gene pair, but uses other functional similarity information to complement interaction data for a better identification of modules.

## Methods

**Figure 1:**
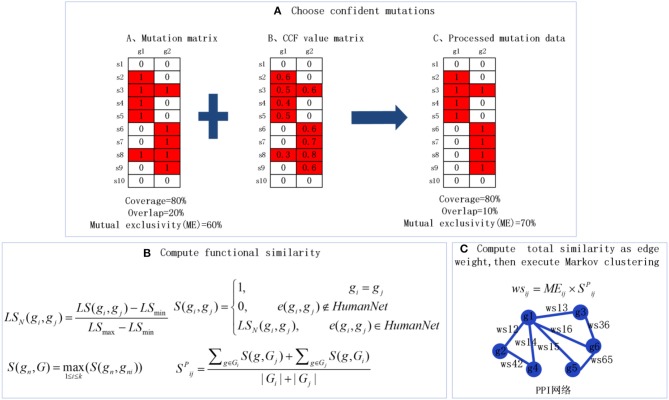
The overview of MCLCluster. **(A)** Integrate CCF data to choose stronger signals and more confident mutations, and compute the mutex of each gene pairs. **(B)** The weighted gene functional interaction network is used to quantify the gene functional similarity in PPI. **(C)** Compute total similarity as edge weight, then execute Markov clustering to extract candidate module.

The identification of the cancer driver modules based on graph clustering (MCLCluster) is introduced in detail. The schematic flowchart is shown in Figure 1.

### Datasets

GBM and BRCA datasets which including CNVs and SNVs mutational data are used for testing, which are downloaded from cBioPortal (Cerami et al., 2012). The GBM dataset contains 550 samples, 1,376 mutant genes, and the BRCA dataset contains 1078 samples, and 1463 mutant genes. We combine non-binary data (CCF) to provide more information and prioritize more important mutations (ie, earlier mutations with larger CCF values). The CCF value indicates the proportion of cancer cells in the mutant sample. CCF data is extracted from read count data (Roth et al., 2014). PPI network are derived from Multinet (Khurana et al., 2013), which contains 109599 interactions between 14445 genes.

In order to verify the reliability, we produce various simulation data with random passenger rate and background noise, and the execution of the entire simulation process use the algorithm in RME. MCLCluster is compared with Multi-Dendrix, FSME Cluster and RME in simulation data. Each simulation datasets contains 500 patients and 200 mutant genes. Mutation noise is achieved by converting a value with opposite values (0 for 1 or 1 for 0) in different probability ranges of 0.05 to 0.11. The remaining genes are considered to be passenger genes and the probability of their mutation uses empirical values.

### Similarity Measure

In order to consider the module property on mutex, functional similarity and connectivity in the PPI network, and to facilitate subsequent graph clustering, we define the edge weights of the PPI network as the product of mutex and functional similarity between gene pair.

#### Functional Similarity

Actually, most of the existing methods widely use cosine coefficient to measure the functional similarity between entities in PPI network, which only consider the network structure and it is too simple to as a functional similarity measurement. So we develop a new metric to measure the entities similarity in PPI with the help of the **w**eighted **g**ene **f**unctional **i**nteraction **n**etwork (**wgfin**), which is downloaded from HumanNet. We use the correlated log-likelihood scores (LS) as a metric of the interaction strength between any two genes in **wgfin**. *LS*_*N*_(*g*_*i*_, *g*_*j*_) represents the normalized value between gene *i* and gene *j* when *LS*(*g*_*i*_, *g*_*j*_) is normalized using min-max normalization, the detail is:

(1) $$\begin{array}{l} {\;LS}_{N}\left(g_{i},g_{j}\right)=\frac{LS\left(g_{i},g_{j}\right)-{LS}_{min}}{{LS}_{max}-{LS}_{min}} \end{array}$$

Here *LS*_*min*_ denotes the minimal *LS* and *LS*_*max*_ denotes the maximal *LS* in **wgfin**. As a result, the similarity *S*(*g*_*i*_, *g*_*j*_) between any two genes that have edges in **wgfin** is calculated:

(2) $$\begin{array}{l} S\left(g_{i},g_{j}\right)=\left\{ \begin{array}{ll} 1, & g_{i}=g_{j}\\ 0, & e\left(g_{i},g_{j}\right)\notin HumanNet\\ {LS}_{N}\left(g_{i},g_{j}\right), & \left(g_{i},g_{j}\right)\in HumanNet\; \end{array}\right. \end{array}$$

Here *e*(*g*_*i*_, *g*_*j*_) represents the edge between gene *i* and gene *j*. Then, the similarity of gene *g*_*n*_ and gene set *G* = {*g*_*n*1_, *g*_*n*2_, …, *g*_*np*_} is calculated as follows:

(3) $$\begin{array}{l} S\left(g_{n},G\right)={max}_{1\leq i\leq p}\left(S\left(g_{n},g_{ni}\right)\right) \end{array}$$

At last, according to the BMA (Best-Match Average) method (Wang et al., 2007; Xiao et al., 2018), the functional similarity of *pg*_*i*_ and *pg*_*j*_ in the PPI network is defined. The detail is as follows:

(4) $$\begin{array}{l} S_{ij}^{P}=\frac{\sum_{g\in G_{i}}S\left(g,G_{j}\right)+\sum_{g\in G_{j}}S\left(g,G_{i}\right)}{\left|G_{i}|+|G_{j}\right|} \end{array}$$

Here *G*_*i*_ and *G*_*j*_ respectively denote the a set of gene connected to *pg*_*i*_ and *pg*_*j*_, and |*G*| denotes the number of genes in *G*.

#### Mutual Exclusivity (Mutex)

To choose stronger signals and more confident mutations, we combine the CCF matrix to process somatic mutation. For each gene, we perform two operations, the one is to delete the mutation with the lowest CCF value, and the other is to delete one mutation when the CCF difference between the two mutations is less than a certain parameter ε (obtain through multiple experiments, usually small than coverage). In this paper, overall consider weighing algorithm efficiency and number of modules, we set the parameter ε = 0.1. The somatic mutation matrix *A* is filtered by CCF matrix, then it will be used to compute mutex, and the detail of each entry is listed as:

(5) $$\begin{array}{l} A_{ab}=\left\{ \begin{array}{ll} 1, & if\;sample\;a\;mutated\;in\;a\;gene\;b\;and\;it\;CCF\\ & value\;meet\;condition\\ 0, & otherwise \end{array}\right. \end{array}$$

In general, mutations between member genes in a driver module appear to be mutually exclusive. The previous work (Vandin et al., 2011) proposed that a pathway or module is a group of genes characterized by high coverage and low coverage overlap. Coverage represents the patient proportion with at least one gene mutation in a group of gene, and coverage overlap is equal to the patient proportion with more than two gene mutations in a group of gene. The mutex is expressed as:

(6) $$\begin{array}{l} ME\left(se\right)=C\left(se\right)\;-\;O\left(se\right) \end{array}$$

Where *ME* denotes mutex, *se* denotes the genes sets, *C* denotes coverage and *O* denotes coverage overlap. Here, we calculate the pairwise and group mutex. Pairwise mutex genes help identify all gene pairs which are may take part in the same module, and the group mutex is applied to compute the mutex of all genes in one module. An example in Figure 1A shows the computation of coverage, coverage overlap and mutex.

Then combine these two properties (functional similarity and mutex) to calculate the total similarity as the edge weight of the PPI network:

(7) $$\begin{array}{l} ws\left({pg}_{i},{pg}_{j}\right)=ME\left({pg}_{i},{pg}_{j}\right)\;\times \;S_{{pg}_{i}{pg}_{j}\;}^{P} \end{array}$$

### Candidate Module Extraction

Here, we apply Markov clustering (MCL) to identify clusters in the PPI network appling the total similarity matrix *ws* derived by Equation (7). Markov clustering is an effective biological network clustering algorithm, which is widely used for the identification of functional modules (Brohee and van Helden, 2006; Vlasblom and Wodak, 2009; Shih and Parthasarathy, 2012). After executing the clustering, closely functional related genes will be grouped into the same cliques, which are as candidate modules and will be used for follow-up modules refinement.

The *GR* = (*N*_*p*_, ϵ_*p*_) denotes the undirected graph in the PPI network, in which *N*_*p*_ represents node sets and ϵ_*p*_ represents edge set. *pg*_*i*_ ∈ *N*_*p*_ represents the *i*-th gene, and *ws*(*pg*_*i*_, *pg*_*j*_) is the edge weight of (*pg*_*i*_, *pg*_*j*_), *ws*(*pg*_*i*_, *pg*_*j*_) > 0 indicate that *pg*_*i*_ interact with *pg*_*j*_ in the PPI network, *ws* (*pg*_*i*_, *pg*_*j*_) = 0 indicate they are not interaction. $P\in {\mathbb{R}}^{\left|N_{p}|\times |N_{p}\right|}$ denotes *GR*′*s* adjacency matrix, the initialization of *P* is:

(8) $$\begin{array}{l} P\left(i,j\right)=\left\{ \begin{array}{cc} ws\left({pg}_{i},{pg}_{j}\right) & \text{if}\;\left({pg}_{i},{pg}_{j}\right)\in \epsilon_{p}\\ \text{ws}({pg}_{i},{pg}_{k}) & if\;\left({pg}_{i}={pg}_{j}\right)\\ 0 & \text{otherwise} \end{array}\right.\;,\;k\in \left[1,|N_{p}|\right] \end{array}$$

The matrix *p* can holds the transition probabilities of the Markov chain defined on *GR*. *p*(*i, j*) denotes the transition probability from *pg*_*i*_ to *pg*_*j*_. Normalize the matrix *P* as follow:

(9) $$\begin{array}{l} \tilde{P}\left(i,j\right)=\frac{P\left(i,j\right)}{\sum_{k=1}^{\left|N_{p}\right|}p\left(k,j\right)} \end{array}$$

Markov clustering contains two processes, which are known as ‘Expand' and ‘Inflate'. When execute the operation process, the ‘Expand' and ‘Inflate' respectively are iteratively assigned to the stochastic matrix. The calculation formula of the Expand operation is:

(10) $$\begin{array}{l} P_{\text{exp}}=\tilde{P}*\tilde{P} \end{array}$$

The inflation parameter *rp* is used in Inflate process to raise each entry in the matrix $\tilde{p}$. The Inflate process can expand the unevenness of each column. That is to say, flows increase where they are already powerful and decrease when they are weak. The Inflate process is expressed like Equation (9):

(11) $$\begin{array}{l} P_{\text{inf}}\left(i,j\right)=\frac{{\tilde{P}\left(i,j\right)}^{rp}}{\sum_{k=1}^{\left|N_{\text{p}}\right|}{\tilde{P}\left(k,j\right)}^{rp}} \end{array}$$

Markov clustering starts from the matrix **P**, and iteratively uses the Expand and Inflate until convergence. After convergence, there is one non-zero value in each column of the final matrix, and those non-zero value in the same row form a node cluster, we can get them as the candidate modules.

### Modules Refinement and Mutex Significant Test

Not all of the clusters (candidate driver modules) obtained by graph clustering can be used as driver modules, nor are all genes in a population members of the module, because it is difficult to obtain the exact size of the module number. Therefore, perform the permutation test on each cluster to evaluate the importance of mutex. However, testing only on the largest cluster may result in the loss of potential subgroups which may pass the test. In order to solve this problem, (Ciriello et al., 2012) proposed the following steps to filter the genes and compute the mutex of the subgroups while limiting the subgroup size. Given a candidate module *C* containing the *r* gene, if a significant *p* value is observed, we will retain the module *C*, and not consider compute the mutex of all its subgroups. Or else, we list all subgroups of *r-*1 size, for each member belongs to the *C*, and executes a permutation test on each subgroup to get a *p* value. It repeats recursively until one of these two conditions is met (Ciriello et al., 2012): a subgroup is significantly mutually exclusive or *r* = 3 (*min_module_size* is 3). After testing, only the cluster that gets the most significant *p* value is reserved as the driver module.

### Evaluating Performance

To compare the performance, F1 score is used for evaluating the power of the identification module. F1 score expressed the trade-off between accuracy (abbreviated to Pr) and recall (abbreviated to Re), which can be computed using true positive (abbreviated to TP), false positive (abbreviated to FP), and false negative (abbreviated to FN). The details are:

(12) $$\begin{array}{l} Pr=\frac{TP}{\left(TP+FP\right)},\;Re=\frac{TP}{\left(TP+FN\right)},\;F1=\frac{2•Pr•Re}{Pr+Re} \end{array}$$

## Results

### GBM

**Figure 2:**
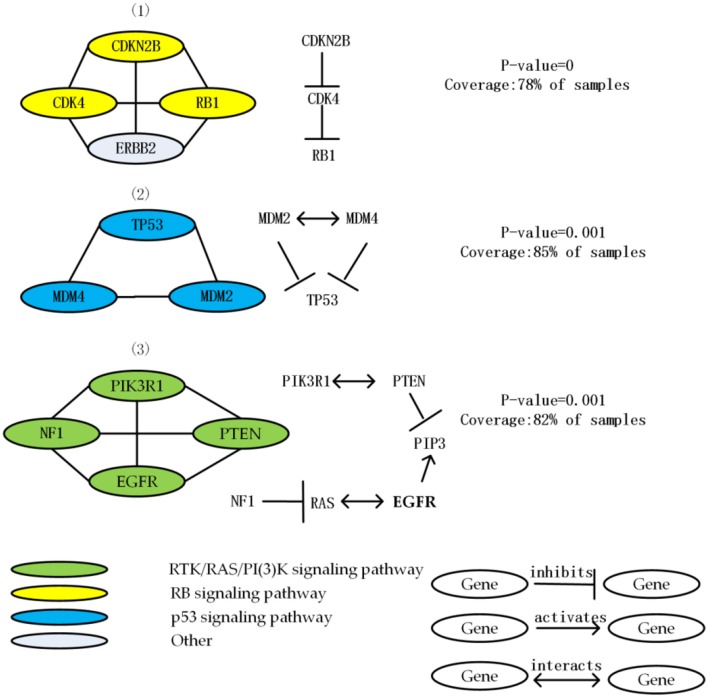
List 3 driver module and the interaction among genes in each driver module in the GBM data. Node color shows the role of GBM in different signal pathways.

We apply MCLCluster to GBM dataset, 3 important driver modules are identified, the detailed information of them are listed in Table 1. The interaction among genes within GBM modules are list in Figure 2. All the genes in these 3 modules are well-known in the GBM research, they are members of the 3 important signaling pathways and their mutation samples are more than five percent.

**Table 1:** Results of GBM.

**No**	**Driver modules**	**Gene number**	**ME (Exclusivity)**	***P*-value**	***ws***
1	CDKN2B CDK4 RB1 ERBB2	4	76%	0	0.834
2	TP53 MDM2 MDM4	3	82%	0.001	0.766
3	PTEN PIK3R1 NF1 EGFR	4	78%	0.001	0.741

*ws is the average value of ws (The sum of the similarities between the pairs divided by the gene number), and ws is the total similarity calculated by Equation (7)*.

The first module contains the mutation of ERBB2, CDK4, and CDKN2B, RB1. The mutation of these four genes cover 78% of the samples, and average functional similarity is 0.834, indicate that the genes in module have similar function. The *p-value* calculated by the permutation test is equal to 0, indicate that the module has significant mutex. Three of these genes (except ERBB2) are from the RB signaling pathway that related to G1/S progression. CDKN2B inhibits CDK4, CDK4 inhibits RB1. CDKN2B and RB1 are core members of the cell cycle and cell cycle mitosis, the over expression of ERBB2 made the proliferation activation, and CDK4 has a strong interaction as a negative regulator of normal cell proliferation (Porta-Pardo et al., 2015; Tang et al., 2016).

The second module includes the mutation of MDM2, MDM4 and TP53. Most of the MDM2 mutation is amplified in the sample. TP53 is an important tumor suppressor gene which is the most common mutant gene in GBM samples. The module is mutated in 85% of the samples, the mutex of the module is 82%, and average functional similarity is 0.766, indicate that the genes in module have similar function, the *p-value* calculated by the permutation test is equal to 0.001, indicate that the module has significant mutex. All the members of this module are well-known members of the p53 signaling pathway (Kim et al., 2015), which is a key and frequently mutated pathway in GBM related to aging and apoptosis (Ciriello et al., 2012). This module contains 3 mutually exclusive gene pairs (all of which are significant), and no gene pair mutates simultaneously (Babur et al., 2015).

The third module consists of deletion of PTEN, the mutation of PIK3R1, NF1, and EGFR. Deletions in PTEN have been linked to the proneural subtype of GBM. Mutations in EGFR and NF1 related to the classical GBM subtype, in addition to the PIK3R1 appearing in the GBM pathway of (Greenman et al., 2007), it has been previously reported to be related to many human cancers (Vandin et al., 2012). The module is mutated in 82% of the samples, the mutex of the module is 78%, and average functional similarity is 0.741, indicate that the genes in module have similar function, the *p-value* calculated by the permutation test is equal to 0.001, indicate that the module has significant mutex. All the members of this module are core members of RTK/RAS/PI(3)K signaling pathway.

### BRCA

**Figure 3:**
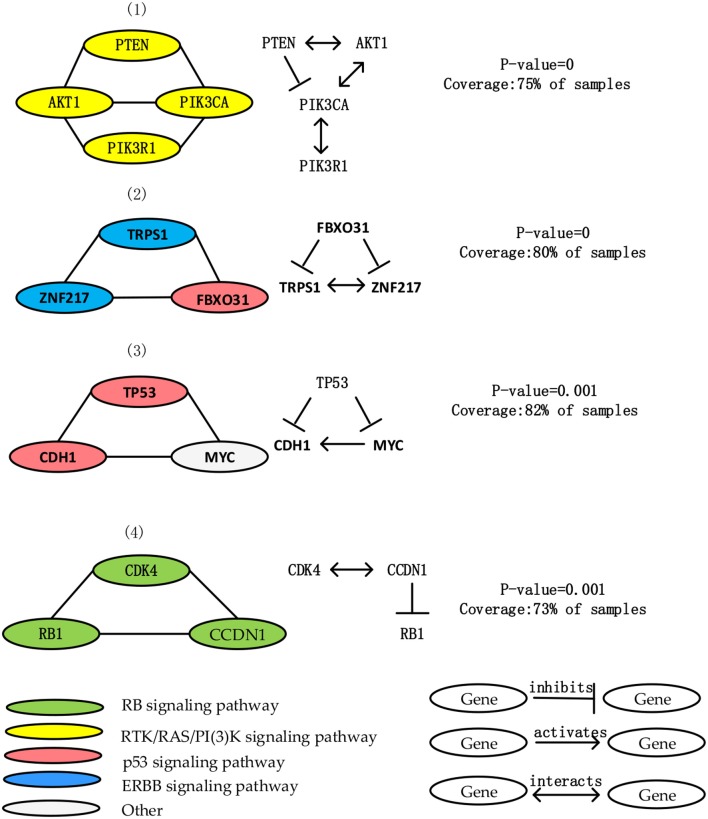
List 4 driver module and the interaction among genes in each driver module in the BRCA data. Node color shows the important role of BRCA in different signal pathways.

We apply MCLCluster to BRCA dataset, 4 driver modules are identified, the detailed information of them are listed in Table 2. The interaction among genes within BRCA modules are list in Figure 3. Most of the genes in these 4 modules are core members of the 4 signaling pathways (p53 signaling, PI(3)K/AKT signaling, ERBB signaling pathway and RB signaling pathway). They are well-known in the BRCA research and their mutation samples are more than five percent. Compared with GBM, these 4 modules cover a smaller percentage of samples, indicate that the mutation heterogeneity or disease heterogeneity of the breast cancer dataset is greater.

**Table 2:** Results of BRCA.

**No**	**Driver modules**	**Gene number**	**ME (Exclusivity)**	***P*-value**	***ws***
1	PTEN PIK3CA PIK3R1 AKT1	4	72%	0	0.824
2	TRPS1 ZNF217 FBXO31	3	74%	0	0.811
3	TP53 CDH1 MYC	3	80%	0.001	0.721
4	FBXO31 RB1 CCDN1	3	70%	0.001	0.714

*ws is the average value of ws (The sum of the similarities between the pairs divided by the gene number), and ws is the total similarity computed by Equation (7)*.

The first module contains the mutation of PIK3CA, PIK3R1, AKT1, PTEN. The mutation of these four genes cover 75% samples, and average functional similarity is 0.824, indicate that the genes in module have similar function. The *p-value* calculated by the permutation test is equal to 0, suggesting that the module has significant mutex. All genes in this module are core members of PI(3)K/AKT signaling pathway. AKT1 interact with PTEN, PIK3R1, and PIK3CA, PTEN inhibits PIK3CA and PIK3R1 (Wu et al., 2015; Mandal and Ma, 2016).

The second module includes TRPS1, ZNF217 and FBXO31 gene mutations. The mutation of these 3 genes cover 89% samples, and average functional similarity is 0.811, indicate that the genes in module have similar function. The p-value calculated by the permutation test is equal to 0, suggesting that the module has significant mutex Two third of genes are members of the ERBB signaling pathway, which is an important breast cancer-related pathway. TRPS1 is a common oncogene that plays an important role in controlling cell cycle during breast cancer (Wu et al., 2014). ZNF217 is proved to be a central role in cancer development, and FBXO31 is proved to be a candidate tumor suppressor gene, by generating Skp Cullin F-box containing SCF complex, it causes cell senescence and has consistent tumor suppressor attributes (Kumar et al., 2005). FBXO31 inhibits TRPS1 and ZNF217.

The third module contains mutations in TP53, CDH1, MYC. The mutation of these 3 genes cover 82% samples, and average functional similarity is 0.721, indicate that the genes in module have similar function. The *p-value* calculated by the permutation test is equal to 0.001, suggesting that the module has significant mutex. Two third of genes are core members of the p53 signaling pathway. Loss or down-regulation of the Ecadherin gene CDH1 at 16q22.1 is associated with breast cancer proliferation and invasion, MYC is an effective tumorigenic activator, a transcription factor, and a key regulator of cell growth, differentiation, and apoptosis (Amgalan and Lee, 2015; Nangalia et al., 2015).

The forth module contains mutations in CCND1, RB1 and CDK4. The mutation of these three genes cover 73% samples, and average functional similarity is 0.714, indicate that the genes in module have similar function. The *p-val*ue calculated by the permutation test is equal to 0.001, suggesting that the module has significant mutex. All of genes in this module are important members of the RB signaling pathway. CDK4 interacts with CCND1, CCND1 inhibits RB1. CCND1 and RB1 encode interact proteins that have an important effect in cell cycle (Placke et al., 2014). CCND1 encodes the cyclind1 protein, it affect the retinoblastoma protein which encoded through overphosphorylation by RB1 (Rozenchan et al., 2014). Hyperphosphorylation of RB inactivates its role as a tumor suppressor gene, so mutations targeting CCND1 or RB1 are of great significance for tumor proliferation (Salgia et al., 2017).

### Simulated Data

#### Identifying Top One Module

To comparing the four methods (MCLCluster, Multi-Dendrix, FSME Cluster and RME), we generated simulation samples considering two parameters (passenger rate and background noise). The Multi-Dendrix need to input the module size, and it is difficult to obtain, so considering fairness, Multi-Dendrix is applied three times for each data set, the module sizes are set to three, four, and five, respectively. The remaining parameter used in other three approaches is set to the default value. By default, MCLCluster will identify multiple modules, the module with the highest *ws* and the lowest *p-value* will be selected. It's worth noting that in simulation experiment, we cannot consider the CCF value.

**Figure 4:**
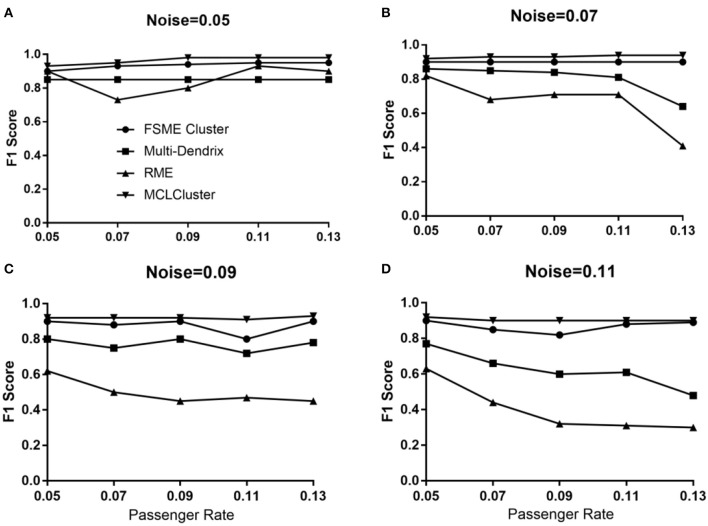
The F1 score of MCLCluster, Multi-Dendrix, FSME Cluster and RME in simulation data for 1 module. **(A)** When noise = 0.05, the F1 score of the four methods with different passenger rate. **(B)** When noise = 0.07, the F1 score of the four methods with different passenger rate. **(C)** When noise = 0.09, the F1 score of the four methods with different passenger rate. **(D)** When noise = 0.11, the F1 score of the four methods with different passenger rate.

As shown in Figure 4, when the noise is 0.05, the four methods all achieve high F1 score under different passenger rates. Among them, MCLCluster received F1 scores above 0.94. In general, when the noise is greater than 0.07, the F1 scores decrease with the increase of passenger rate in Multi-Dendrix and RME. In addition, when noise and passenger rates all greater than or equal to 0.09, the F1 scores of RME are all less than 0.6. MCLCluster and FSME Cluster also faces a decline in F1 score, when the noise is greater than 0.09. MCLCluster have better performance than the others in all cases, which shows that MCLCluster have a strong ability to detect mutually exclusive drive modules. Compared with the other three methods, under different noise environments, as the passenger rate increases, the MCLCluster shows good stability.

#### Identifying Multiply Modules

**Figure 5:**
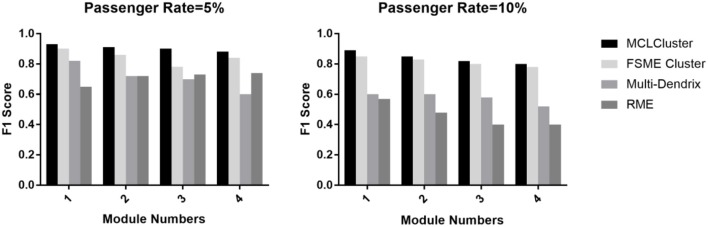
The F1 score of MCLCluster, Multi-Dendrix, FSME Cluster and RME in simulation data for multiply modules.

We identify one to four modules to compare MCLCluster, Multi-Dendrix, FSME Cluster and RME. The passenger rate is set to 0.05 and 0.10, and the module noise is set to 0.10. We can see from Figure 5, the F1 scores of the four methods have a slight downward trend. When the passenger rate is 0.05, the RME showed a high F1 score relative to Multi-Dendrix in most cases, and when the passenger mutation rate increased to 0.10, Multi-Dendrix performed better than RME. The MCLCluster can outperform all other methods in any cases, both the increased module numbers and the two different passenger rates.

## Conclusions and Discussions

We develop a new approach named MCLCluster, which uses somatic mutation data, Cancer Cell Fraction (CCF) data, gene functional interaction network and protein-protein interaction (PPI) network to detect multiple driver modules that simultaneously display functional similarity and mutation mutex in cancer. The reliability of MCLCluster is verified using GBM and BRCA cancer datasets and simulation samples. Taking GBM as an example, MCLCluster successfully identified 3 driver modules, which include some important and common driver genes, like CDKN2B, CDK4, RB1, ERBB2, TP53, EGFR etc., which provided important verification for this method. In the simulation dataset, the MCLCluster can maintain higher performance than Multi-Dendrix, FSME Cluster and RME in F1 scores. With the increase of noise, passenger rate and the module numbers in the simulation data, our method keeps a stable and sufficiently high F1 score, indicate that the MCLCluster can accurately identify modules in complex cases. BRCA and GBM are used as examples to prove the effectiveness of the method, and actually it is universal and can be applied to other type of interest cancer. In this paper, we use a general method to preprocess the real data set and construct the simulated data set, which is a feasible method verified by a lot of experiments. In addition, some parts of our method are general and can be used to solve other bioinformatics problems, such as the similarity measure method, which can be used to identify cancer-related microRNA modules based on microRNA-disease associations.

However, like previous researches of Multi-Dendrix, FSME Cluster and RME, MCLCluster is also designed for large sample sets to achieve statistical significance. Therefore, applying MCLCluster to a small number of samples may have some limitations. Some extensions can be used to further improve the MCLCluster method, for example, we can integrate the methylation and mRNA expression data, and use well-researched pathways reported in many literatures as a priori information. As the genome sequencing dataset in TCGA expands to more than 20 types of cancer, MCLCluster will be an important approach to identify new driver modules in different cancer.

## Data Availability Statement

Publicly available datasets were analyzed in this study. This data can be found here: https://www.cancer.gov/about-nci/organization/ccg/research/structural-genomics/tcga.

## Author Contributions

LW conceived the study and supervised the study. WZ and YL developed the method. YL and YZ implemented the algorithms. WZ and YZ analyzed the data. WZ and LW wrote the manuscript. YC reviewed and improved the manuscript. All authors read and approved the final manuscript.

### Conflict of Interest

The authors declare that the research was conducted in the absence of any commercial or financial relationships that could be construed as a potential conflict of interest.

## References

[B1] Ahmed R. Baali I. Erten C. Hoxha E. Kazan H. (2019). MEXCOWalk:Mutual Exclusion and Coverage Based Random Walk to Identify Cancer Modules. Bioinformatics 36, 872–879. 10.1093/bioinformatics/btz655 31432076

[B2] Amgalan B. Lee H. (2015). DEOD: uncovering dominant effects of cancer-driver genes based on a partial covariance selection method. Bioinformatics 31, 52–60. 10.1093/bioinformatics/btv175 25819079

[B3] Babaei S. Hulsman M. Reinders M. de Ridder J. (2013). Detecting recurrent gene mutation in interaction network context using multi-scale graph diffusion. BMC Bioinform. 14, 345–357. 10.1186/1471-2105-14-29 23343428 PMC3626877

[B4] Babur O. Gonen M. Aksoy B. A. Schultz N. Ciriello G. Sander C.. (2015). Systematic identification of cancer driving signaling pathways based on mutual exclusivity of genomic alterations. Genome Biol. 16, 34–45. 10.1186/s13059-015-0612-6 25887147 PMC4381444

[B5] Brohee S. van Helden J. (2006). Evaluation of clustering algorithms for protein-protein interaction networks. Bmc Bioinformatics. 7, 2944–2952. 10.1186/1471-2105-7-488 17087821 PMC1637120

[B6] Cancer Genome Atlas Research N. (2008). Comprehensive genomic characterization defines human glioblastoma genes and core pathways. Nature 455, 1061–1068. 10.1038/nature07385 18772890 PMC2671642

[B7] Cerami E. Gao J. Dogrusoz U. Gross B. E. Sumer S. O. Aksoy B. A.. (2012). The cBio cancer genomics portal: an open platform for exploring multidimensional cancer genomics data. Cancer Discov. 2, 401–404. 10.1158/2159-8290.CD-12-0095 22588877 PMC3956037

[B8] Ciriello G. Cerami E. Sander C. Schultz N. (2012). Mutual exclusivity analysis identifies oncogenic network modules. Genome Res. 22, 398–406. 10.1101/gr.125567.111 21908773 PMC3266046

[B9] Dao P. Kim Y. A. Wojtowicz D. Madan S. Sharan R. Przytycka T. M. (2017). Bewith: a between-within method to discover relationships between cancer modules via integrated analysis of mutual exclusivity, co-occurrence and functional interactions. PLoS Comput. Biol. 13:e1005695. 10.1371/journal.pcbi.1005695 29023534 PMC5638227

[B10] Dees N. D. Zhang Q. Y. Kandoth C. Wendl M. C. Schierding W. Koboldt D. C.. (2012). MuSiC: Identifying mutational significance in cancer genomes. Genome Res. 22, 1589–1598. 10.1101/gr.134635.111 22759861 PMC3409272

[B11] Deng Y. L. Luo S. Y. Deng C. Y. Luo T. Yin W. K. Zhang H. Y.. (2019). Identifying mutual exclusivity across cancer genomes: computational approaches to discover genetic interaction and reveal tumor vulnerability. Brief Bioinform. 20, 254–266. 10.1093/bib/bbx109 28968730

[B12] Greenman C. Stephens P. Smith R. Dalgliesh G. L. Hunter C. Bignell G.. (2007). Patterns of somatic mutation in human cancer genomes. Nature 446, 153–158. 10.1038/nature05610 17344846 PMC2712719

[B13] Hou J. P. Emad A. Puleo G. J. Ma J. Milenkovic O. (2016). A new correlation clustering method for cancer mutation analysis. Bioinformatics 32, 3717–3728. 10.1093/bioinformatics/btw546 27540270 PMC6078169

[B14] Hua X. Xu H. M. Yang Y. N. Zhu J. Liu P. Y. Lu Y. (2013). DrGaP: a powerful tool for identifying driver genes and pathways in cancer sequencing studies. Am. J. Hum. Genet. 93, 439–451. 10.1016/j.ajhg.2013.07.003 23954162 PMC3769934

[B15] Jia P. L. Zhao Z. M. VarWalker. (2014). Personalized mutation network analysis of putative cancer genes from next-generation sequencing data. PLoS Comput. Biol. 10, 342–353. 10.1371/journal.pcbi.1003460 24516372 PMC3916227

[B16] Khurana E. Fu Y. Chen J. M. Gerstein M. (2013). Interpretation of genomic variants using a unified biological network approach. Plos Comput. Biol. 9:e1002886. 10.1371/journal.pcbi.1002886 23505346 PMC3591262

[B17] Kim Y. A. Cho D. Y. Dao P. Przytycka T. M. (2015). MEMCover: integrated analysis of mutual exclusivity and functional network reveals dysregulated pathways across multiple cancer types. Bioinformatics 31, 84–92. 10.1093/bioinformatics/btv247 26072494 PMC4481701

[B18] Kumar R. Neilsen P. M. Crawford J. McKirdy R. Lee J. Powell J. A.. (2005). FBXO31 is the chromosome 16q24.3 senescence gene, a candidate breast tumor suppressor, and a component of an SCF complex. Cancer Res. 65, 11304–11313. 10.1158/0008-5472.CAN-05-0936 16357137

[B19] La Vecchia S. Sebastian C. (2020). Metabolic pathways regulating colorectal cancer initiation and progression. Semin. Cell Dev. Biol. 98, 63–70. 10.1016/j.semcdb.2019.05.018 31129171

[B20] Leiserson M. D. Vandin F. Wu H. T. Dobson J. R. Raphael B. R. (2014). Pan-cancer identification of mutated pathways and protein complexes. Cancer Res. 74, 112–123. 10.1158/1538-7445.AM2014-5324

[B21] Leiserson M. D. M. Blokh D. Sharan R. Raphael B. J. (2013). Simultaneous identification of multiple driver pathways in cancer. PLoS Comput. Biol. 9, 23–34. 10.1371/journal.pcbi.1003054 23717195 PMC3662702

[B22] Leiserson M. D. M. Wu H. T. Vandin F. Raphael B. J. (2015). CoMEt: a statistical approach to identify combinations of mutually exclusive alterations in cancer. Genome Biol. 16:160. 10.1186/s13059-015-0700-7 26253137 PMC4531541

[B23] Liu X. Xi J. Zhang C. Feng H. Li A. Wang M. (2017). Identification of driver network modules in protein-protein interaction network using patient mutation profiles, in 2017. 10th International Congress on Image and Signal Processing, BioMedical Engineering and Informatics (CISP-BMEI) (Shanghai: IEEE), 1–6. 10.1109/CISP-BMEI.2017.8302274

[B24] Mandal B. N. Ma J. (2016). l(1) regularized multiplicative iterative path algorithm for non-negative generalized linear models. Comput. Stat. Data Anal. 101, 289–299. 10.1016/j.csda.2016.03.009

[B25] Nambara S. Kurashige J. Saito T. Komatsu H. Ueda M. Sakimura S. (2015). Omics approach to identify driver genes for peritoneal dissemination of gastric cancer cells. Cancer Res. 75:5169 10.1158/1538-7445.AM2015-5169

[B26] Nangalia J. Nice F. L. Wedge D. C. Godfrey A. L. Grinfeld J. Thakker C.. (2015). DNMT3A mutations occur early or late in patients with myeloproliferative neoplasms and mutation order influences phenotype. Haematologica 100, E438–E442. 10.3324/haematol.2015.129510 26250577 PMC4825297

[B27] Network C. G. A. R. (2012). Comprehensive genomic characterization of squamous cell lung cancers the cancer genome atlas research network. Nature 489, 519–525. 10.1038/nature11666 22960745 PMC3466113

[B28] Paull E. O. Carlin D. E. Niepel M. Sorger P. K. Haussler D. Stuart J. M. (2013). Discovering causal pathways linking genomic events to transcriptional states using tied diffusion through interacting events (TieDIE). Bioinformatics 29, 2757–2564. 10.1093/bioinformatics/btt471 23986566 PMC3799471

[B29] Pelegrina L. T. Sanhueza M. D. Caceres A. R. R. Cuello-Carrion D. Rodriguez C. E. Laconi M. R. (2020). Effect of progesterone and first evidence about allopregnanolone action on the progression of epithelial human ovarian cancer cell lines. J. Steroid Biochem. Mol. Biol. 196:105492. 10.1016/j.jsbmb.2019.105492 31614205

[B30] Placke T. Faber K. Nonami A. Putwain S. L. Salih H. R. Heidel F. H.. (2014). Requirement for CDK6 in MLL-rearranged acute myeloid leukemia. Blood 124, 13–23. 10.1182/blood-2014-02-558114 24764564 PMC4190617

[B31] Porta-Pardo E. Hrabe T. Godzik A. (2015). Cancer3D: understanding cancer mutations through protein structures. Nucleic Acids Res. 43, 968–973. 10.1093/nar/gku1140 25392415 PMC4383948

[B32] Reyna M. A. Leiserson M. D. M. Raphael B. J. (2018). Hierarchical hotnet: identifying hierarchies of altered subnetworks. Bioinformatics 34, 972–980. 10.1093/bioinformatics/bty613 30423088 PMC6129270

[B33] Roth A. Khattra J. Yap D. Wan A. Laks E. Biele J.. (2014). PyClone: statistical inference of clonal population structure in cancer. Nat. Methods. 11, 396–398. 10.1038/nmeth.2883 24633410 PMC4864026

[B34] Rozenchan P. B. Mundim F. G. Roela R. A. Katayama M. L. Pasini F. S. Brentani H. (2014). RHOA, RAC1 and PAK1 evaluation in paired stromal fibroblasts of breast cancer primary and of lymph node metastasis: importance of these biomarkers in lymph node invasion. Cancer Res. 74, 213–224. 10.1158/1538-7445.AM2014-186

[B35] Salgia R. Weaver R. W. McCleod M. Stille J. R. Yan S. B. Roberson S.. (2017). Prognostic and predictive value of circulating tumor cells and CXCR4 expression as biomarkers for a CXCR4 peptide antagonist in combination with carboplatin-etoposide in small cell lung cancer: exploratory analysis of a phase II study. Invest. New Drugs 35, 334–344. 10.1007/s10637-017-0446-z 28299514 PMC5418321

[B36] Shih Y. K. Parthasarathy S. (2012). Identifying functional modules in interaction networks through overlapping Markov clustering. Bioinformatics 28, I473–I479. 10.1093/bioinformatics/bts370 22962469 PMC3436797

[B37] Tang C. Jiang Y. S. Shao W. W. Shi W. Gao X. S. Qin W. Y.. (2016). Abnormal expression of FOSB correlates with tumor progression and poor survival in patients with gastric cancer. Int. J. Oncol. 49, 1489–1496. 10.3892/ijo.2016.3661 27633497

[B38] Tomczak K. Czerwinska P. Wiznerowicz M. (2015). The cancer genome atlas (TCGA): an immeasurable source of knowledge. Contemp Oncol. 19, A68–A77. 10.5114/wo.2014.47136 25691825 PMC4322527

[B39] Vandin F. Upfal E. Raphael B. J. (2011). Algorithms for detecting significantly mutated pathways in cancer. J. Comput. Biol. 18, 507–522. 10.1007/978-3-642-12683-3_33 21385051

[B40] Vandin F. Upfal E. Raphael B. J. (2012). *De novo* discovery of mutated driver pathways in cancer. Genome Res. 22, 375–385. 10.1101/gr.120477.111 21653252 PMC3266044

[B41] Vlasblom J. Wodak S. J. (2009). Markov clustering versus affinity propagation for the partitioning of protein interaction graphs. BMC Bioinformatics 10:99. 10.1186/1471-2105-10-99 19331680 PMC2682798

[B42] Wang J. Zuo Y. Man Y. G. Avital I. Stojadinovic A. Liu M.. (2015). Pathway and network approaches for identification of cancer signature markers from omics data. J. Cancer 6, 54–65. 10.7150/jca.10631 25553089 PMC4278915

[B43] Wang J. Z. Du Z. D. Payattakool R. Yu P. S. Chen C. F. (2007). A new method to measure the semantic similarity of GO terms. Bioinformatics 23, 1274–1281. 10.1093/bioinformatics/btm087 17344234

[B44] Weinstein J. N. Collisson E. A. Mills G. B. Shaw K. R. M. Ozenberger B. A. Ellrott K.. (2013). The cancer genome atlas pan-cancer analysis project. Nat. Genet. 45, 1113–1120. 10.1038/ng.2764 24071849 PMC3919969

[B45] Wood L. D. Parsons D. W. Jones S. Lin J. Sjoblom T. Leary R. J.. (2007). The genomic landscapes of human breast and colorectal cancers. Science 318, 1108–1113. 10.1126/science.1145720 17932254

[B46] Wu H. Gao L. Li F. Song F. Yang X. F. Kasabov N. (2015). Identifying overlapping mutated driver pathways by constructing gene networks in cancer. BMC Bioinformatics. 16, 334–345. 10.1186/1471-2105-16-S5-S3 25859819 PMC4402595

[B47] Wu L. L. Wang Y. Z. Liu Y. Yu S. Y. Xie H. Shi X. J.. (2014). A central role for TRPS1 in the control of cell cycle and cancer development. Oncotarget 5, 7677–7690. 10.18632/oncotarget.2291 25277197 PMC4202153

[B48] Xi J. N. Wang M. H. Li A. (2018). Discovering mutated driver genes through a robust and sparse co-regularized matrix factorization framework with prior information from mRNA expression patterns and interaction network. BMC Bioinform. 19:214. 10.1186/s12859-018-2218-y 29871594 PMC5989443

[B49] Xiao Q. Luo J. W. Liang C. Cai J. Ding P. J. (2018). A graph regularized non-negative matrix factorization method for identifying microRNA-disease associations. Bioinformatics 34, 239–248. 10.1093/bioinformatics/btx545 28968779

[B50] Yang H. Wei Q. Zhong X. Yang H. Li B. (2017). Cancer driver gene discovery through an integrative genomics approach in a non-parametric Bayesian framework. Bioinformatics 33, 483–490. 10.1093/bioinformatics/btw662 27797769 PMC6075201

[B51] Zhang J. Zhang S. (2016). The discovery of mutated driver pathways in cancer: models and algorithms. IEEE/ACM Trans. Comput. Biol. Bioinform. 15, 988–998. 10.1109/TCBB.2016.2640963 28113329

[B52] Zhang W. Wang S. L. (2019a). An integrated framework for identifying mutated driver pathway and cancer progression. Ieee/Acm Trans. Comput. Biol. Bioinform. 16, 455–464. 10.1109/T.C.B.B.2017.2788016 29990286

[B53] Zhang W. Wang S. L. (2019b). A novel method for identifying the potential cancer driver genes based on molecular data integration. Biochem. Genet. 10.1007/s10528-019-09924-2 31115714

[B54] Zhao B. H. Zhao Y. L. Zhang X. X. Zhang Z. H. Zhang F. Wang L. (2019). An iteration method for identifying yeast essential proteins from heterogeneous network. BMC Bioinformatics 20:355. 10.1186/s12859-019-2930-2 31234779 PMC6591974

[B55] Zhao J. Zhang S. Wu L. Y. Zhang X. S. (2012). Efficient methods for identifying mutated driver pathways in cancer. Bioinformatics 28, 2940–2947. 10.1093/bioinformatics/bts564 22982574

[B56] Zheng C. H. Yang W. Chong Y. W. Xia J. F. (2016). Identification of mutated driver pathways in cancer using a multi-objective optimization model. Comput. Biol. Med. 72, 22–29. 10.1016/j.compbiomed.2016.03.002 26995027

